# Supplementary material for: Asymmetric Nature of MscL Opening Revealed by Molecular Dynamics Simulations
Source: J Chem Inf Model. 2025 Jun 5;65(12):6129–43. doi: 10.1021/acs.jcim.5c00307 (PMC12199302; doi:10.1021/acs.jcim.5c00307)
Supplement: Supplementary file 6 [file ci5c00307_si_006.pdf]

Supplementary Information for:

Asymmetric nature of MscL opening revealed by molecular dynamics simulations.

Olga N. Rogacheva[1] *and Wojciech Kopec*[1,2*]

[1]Computational Biomolecular Dynamics Group, Max Planck Institute for Multidisciplinary Sciences, Am Fassberg 11, 37077 Göttingen, Germany

[2]Department of Chemistry, Queen Mary University of London, Mile End Road, London E1 4NS, United Kingdom

*Corresponding author ; w.kopec@qmul.ac.uk

Number of pages: 20

Number of figures: 9

Number of tables: 1

S.1. Comments on the number of replicas

The preliminary concept was to generate a single sequence of transitions for each of the 12 original replicas (as designated in Figure 2a, main text), thereby ensuring that the transitions were entirely uncorrelated. However, there are two exceptions to this. Firstly, the two paths of Replica 7 from the closed state to S1 exhibited such marked differences that we elected to retain both. Secondly, for Replica 12, the initial and subsequent transitions occurred in rapid succession during the first production run (step 4 in Figure 2a, main text). As a result, both paths were processed. Consequently, the initiation of 12 replicas in a closed state resulted in the identification of 14 distinct pathways leading to the S1 state. In the final stage of the simulation (step 5 in Figure 2a, main text), the number of replicas was reduced to the original 12.

S.2. Pre-separation of the closed and S1 state for SVM

As the SVM classifier is a supervised learning method, it was necessary to make an initial determination as to which frames belong to the closed or S1 states. To achieve this, we projected all paths onto the PC1, constructed from the zero-tension contacts (Methods, "Features selection"), and employed a two-component Gaussian mixture model for the differentiation between the closed and S1 states. As the PC1 did not provide a unified transition state region, the Gaussian mixture model was applied to each path separately. The separation was deemed successful if the data did not overlap within a two-sigma interval. However, this criterion was not met for all paths (e. g. the path colored in gray in Figure S1 a, e), necessitating the adoption of an alternative approach. Considered this evidence, we applied PCA to the time series data obtained

for all filtered contacts. The first principal component of the all-contacts PCA was capable of differentiating between the closed and S1 states across all paths (Figure S1 b, f). Accordingly, the all-contacts PC1 was utilized for the classification of the path frames between the closed and S1 states. The classification approach employed was identical to that previously described in this paragraph, including the application of a Gaussian mixture model to each path separately. This was done because the new PC1 also failed to identify a unified transition state region.

S.3. Performance of the SVM-based collective variable

The projections of the paths onto the combined SVM-based collective variable are shown in Figures S1c and g. As illustrated in Figure S1 g, the graph of the configuration densities along the collective variable reveals that the low-density region falls within a narrow range of collective variable values for all paths. It can thus be postulated that this region may correspond to the putative transition state, and that the SVM-based collective variable is capable of differentiating between closed and S1 states. The only deviation from the transition state position common to the majority of the paths was demonstrated by Replica 4 (Figure S1 g, red path). Upon visual examination of this trajectory, it was observed that flexible loops connecting the TM1 and TM2 helices were stuck together at the mouth of the channel (Figure S1 g, inset). Throughout the trajectory, they attempted to disentangle themselves and eventually succeeded. As it was uncertain whether this event would significantly alter the free energy landscape of the MscL opening, this pathway was excluded from further analysis.

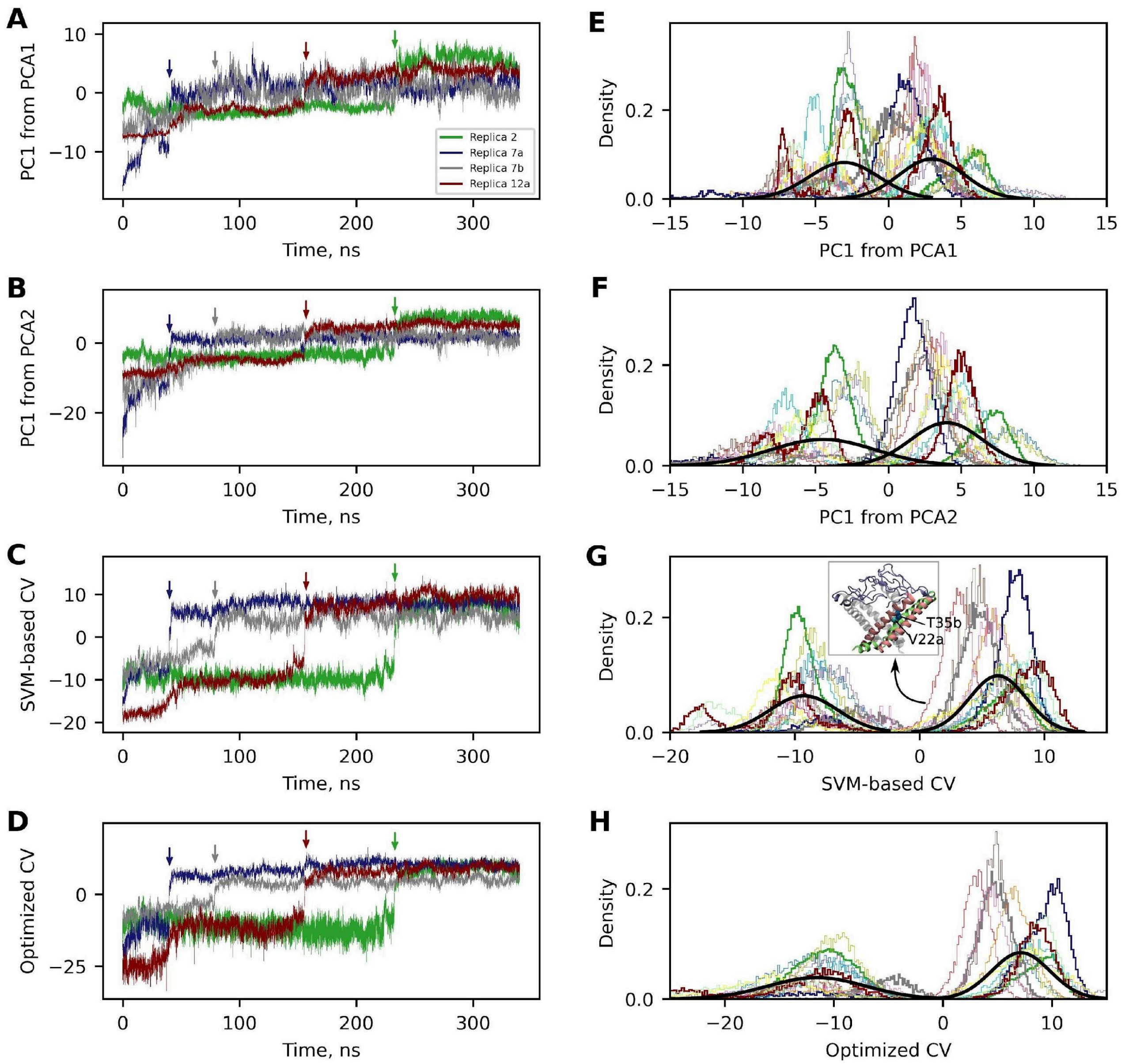

Figure S1. Projections of the simulated paths onto the different collective variables: **A** and **E.** PC1 from PCA, applied to stable contacts at zero tension (termed PCA1); **B** and **F.** PC1 from PCA, applied to a large set of contacts that included protein residues and lipids (termed PCA2); **C** and **G.** SVM-based collective variable, derived as a linear combination of the contact and distance SVM-based collective variables; **D** and **H.** Collective variable optimized using computed commitors. **A-D.** Time series of 4 selected paths (the same paths are highlighted with bold in figures **E-H**), arrows point to the events of transition from the closed to S1 state. **E-H.**

Densities of configurations visited by paths, along the collective variables. Black bold Gaussians show the result of applying a Gaussian mixture model to pooled data from all paths. Inset in panel **G** shows the S1 state for Replica 4 (red path). The conformational transition was undertaken by chain B, which is shown in pink. The adjacent TM1 helix of chain A is indicated in lime. Although the protein is in the S1 state, which is confirmed for example by formation of the critical contact V22a-T35b, its conformation appears to be relatively closed, as the flexible loops (ice-blue) are stuck together at the top of the channel.

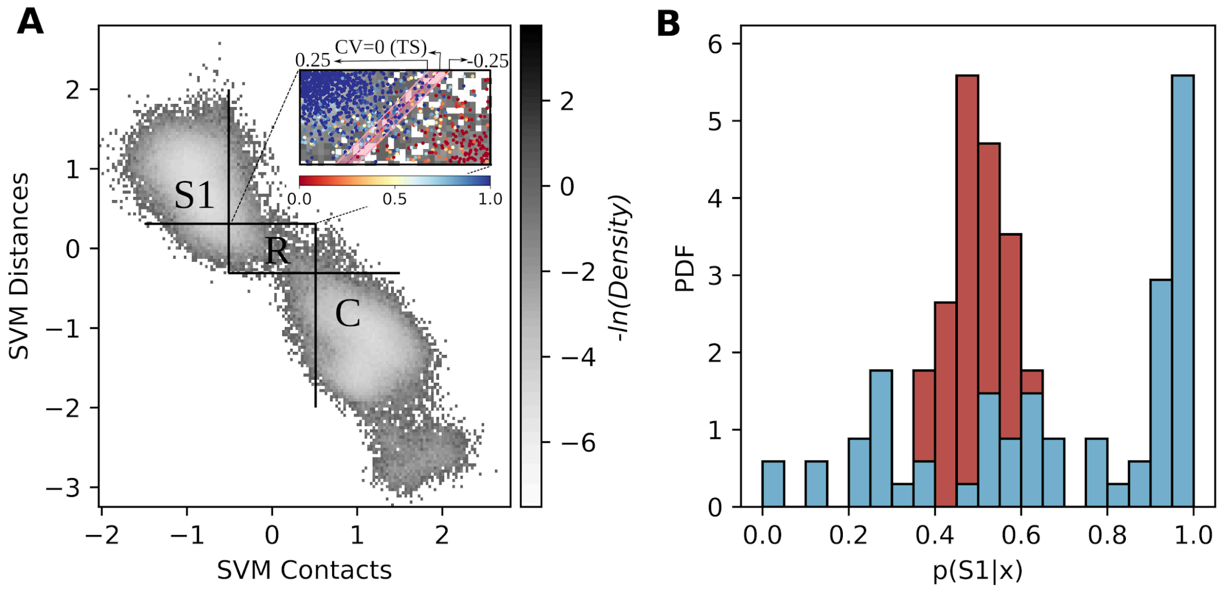

Figure S2. Methodology of the committor analysis. **A.** The gray landscape represents a density of all simulated paths, except replica 4 and replica 11, projected onto the space formed by the SVM-based collective variables. The regions approximating the closed (C) and S1 states and the reactive path region (R) are shown. The snapshots for committor computations were selected from the reactive path region. The inset shows all snapshots colored by the corresponding committor value. The pink dashed line indicates the optimal position of the transition state on the SVM-based collective variable. The pink stripe was introduced as an extension of the transition

state to allow calculating the distribution of committor values in the transition state. **B.** The distribution of committor values in the transition state, defined in the inset of panel a), is shown in blue. A red histogram is given for comparison to show the approximate appearance of the "ideal" committor distribution. It is obtained by taking a sample of the same size as used for the blue histogram from the Gaussian distribution (mean=0.5, σ=0.07).

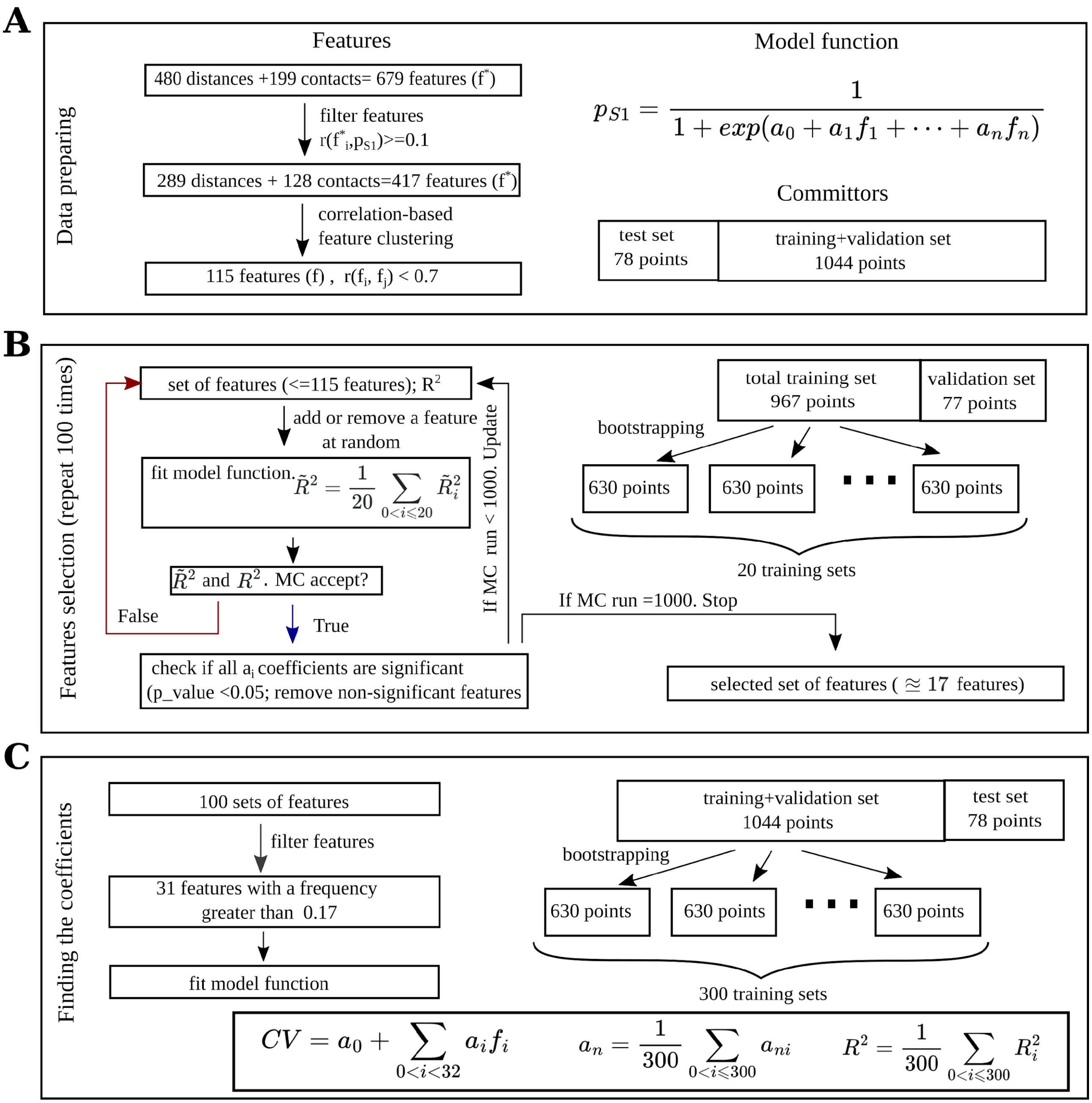

Figure S3. Methodology for the collective variable optimization. **A.** The initial set of features is subjected to elimination of multicollinearity. The total set of committor values is divided into a 78-point test set and a 1044-point remainder set. The form of the model logistic function is shown. **B.** The selection of features is conducted via a Monte Carlo procedure, with the objective of maximizing the adjusted R^2 value. The 1,044-point committor values set is divided into a

validation set and a 967-point set. The latter is resampled 20 times using the bootstrap methodology, resulting in 20 training sets. For each combination of features, the model is fitted twenty times on different training sets, and the mean R^2 value is estimated on the validation set. This process is repeated 100 times. **C.** The 31 features with the highest frequency of occurrence were selected from the optimized combinations of features. The corresponding linear coefficients were then determined via fitting of the model function to committor values. This was achieved by fitting the model 300 times, with 300 training sets. Subsequently, the final adjusted R^2, MSE, and MAE values were calculated on a test set.

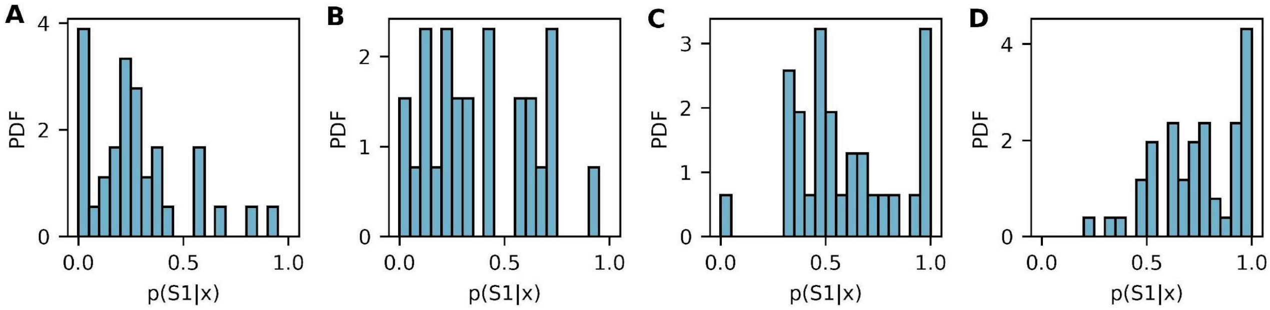

Figure S4. The histograms demonstrate the distributions of the committors for the various values of the optimized collective variable. The collective variable ranges from **A.** -1.25 to -0.75, **B.** -0.75 to -0.25, **C.** -0.25 to 0.25 (transition state), and **D.** 0.25 to 0.75.

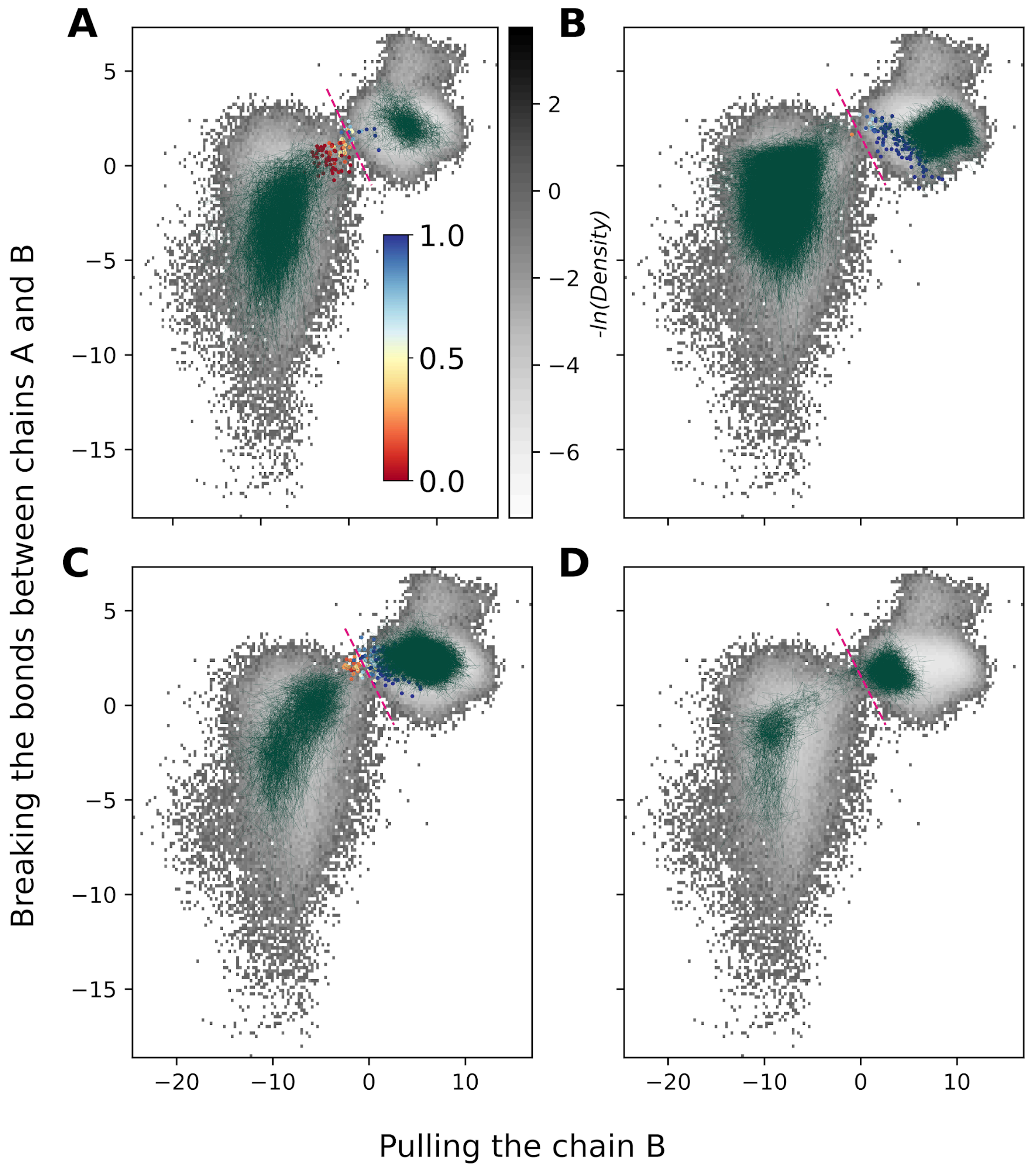

Figure S5. Individual trajectories plotted onto the space formed by the optimized collective variable divided into two parts. The gray landscape represents the density of all simulated paths, with the exception of replicas 4 and 11. All snapshots for which committors were computed are represented by dots colored according to the corresponding committor value. The pink dashed

line indicates the optimal position of the transition state. The trajectories of each replica are displayed on the corresponding panels with a blue-green line: **A.** Replica 1, **B.** Replica 2, **C.** Replica 3, **D.** Replica 4.

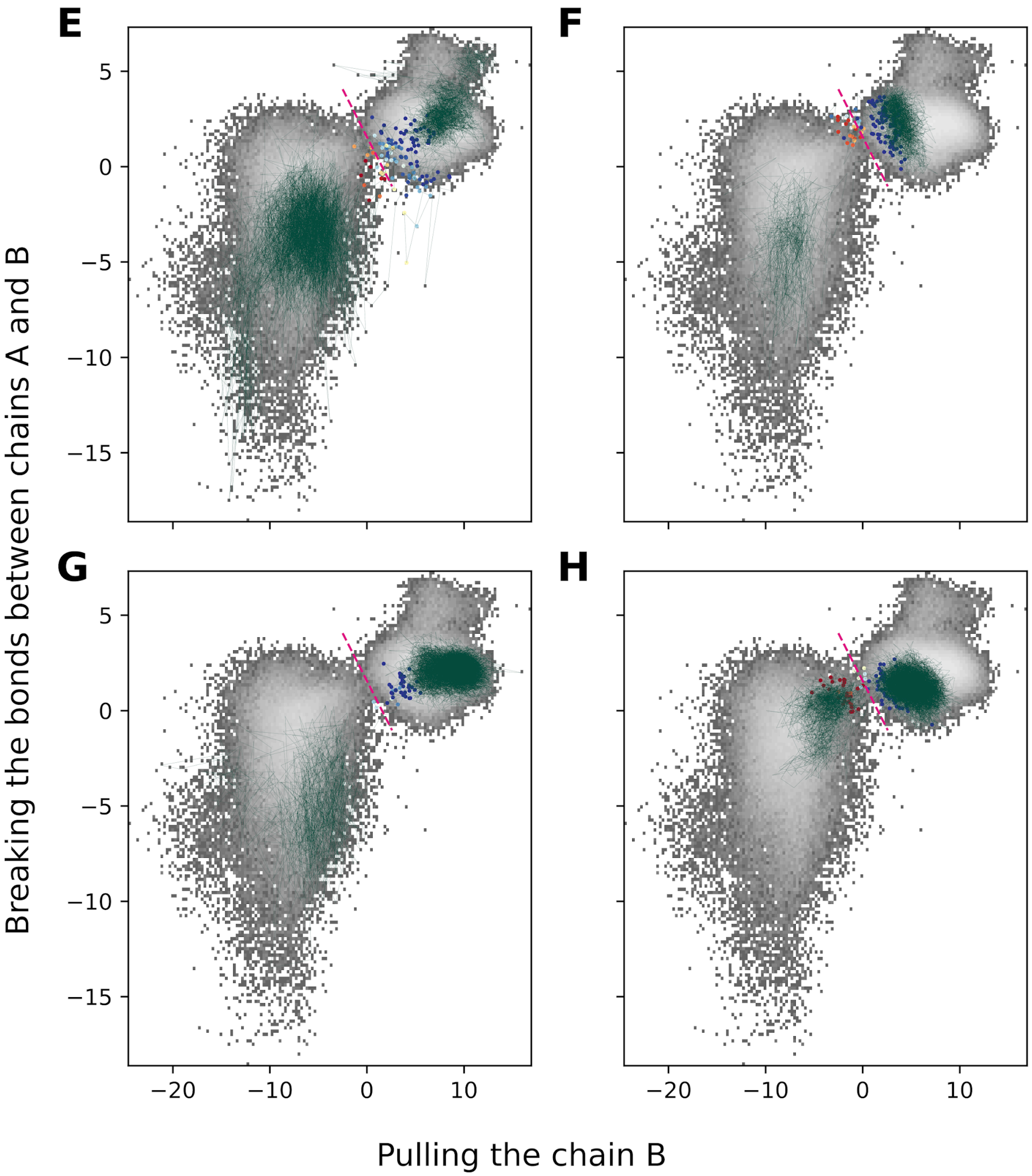

Figure S5. Continued. **E.** Replica 5, **F.** Replica 6, **G.** Replica 7a, **H.** Replica 7b.

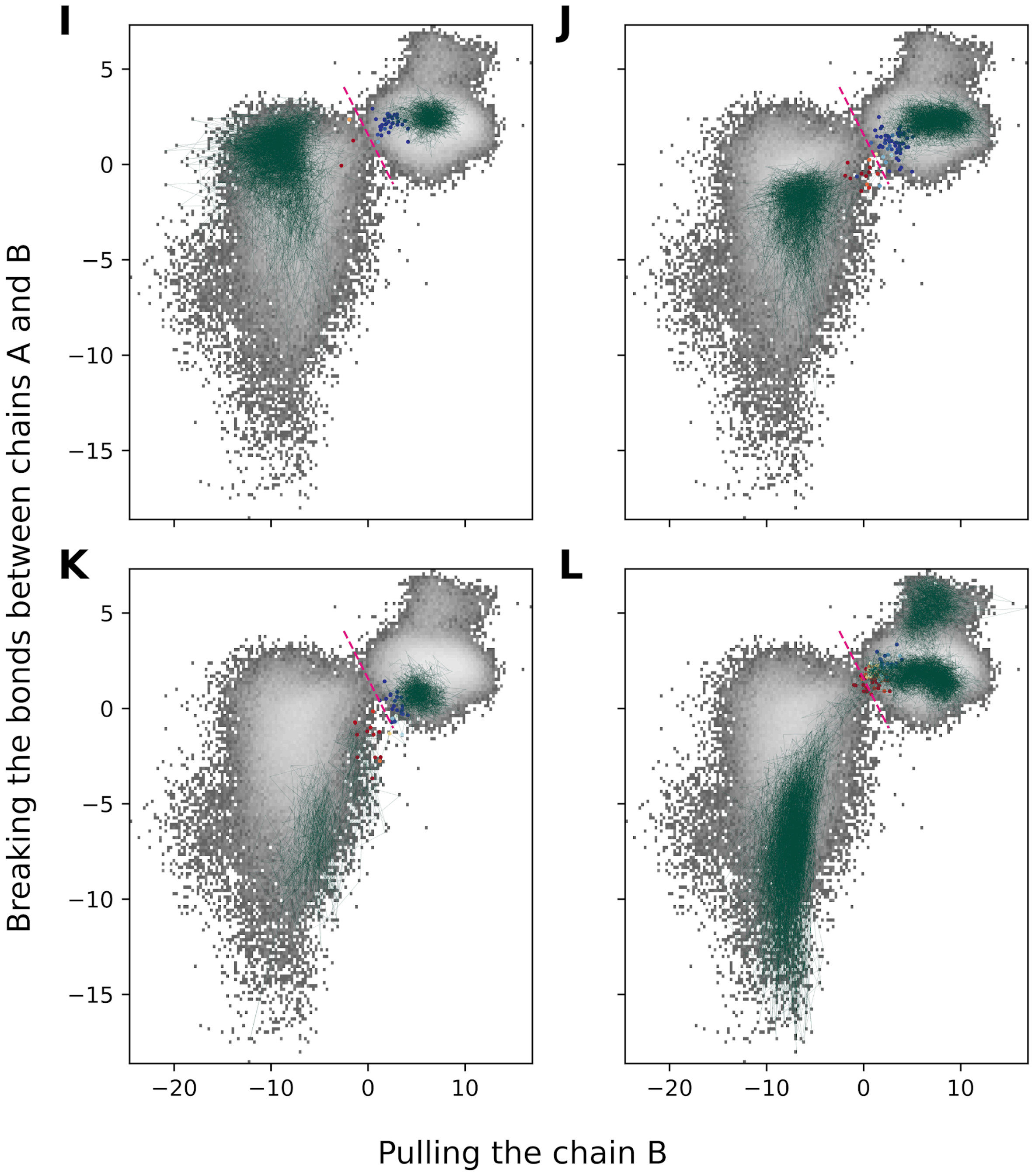

Figure S5. Continued. **I.** Replica 8 **J.** Replica 9, **K.** Replica 10, **L.** Replica 11.

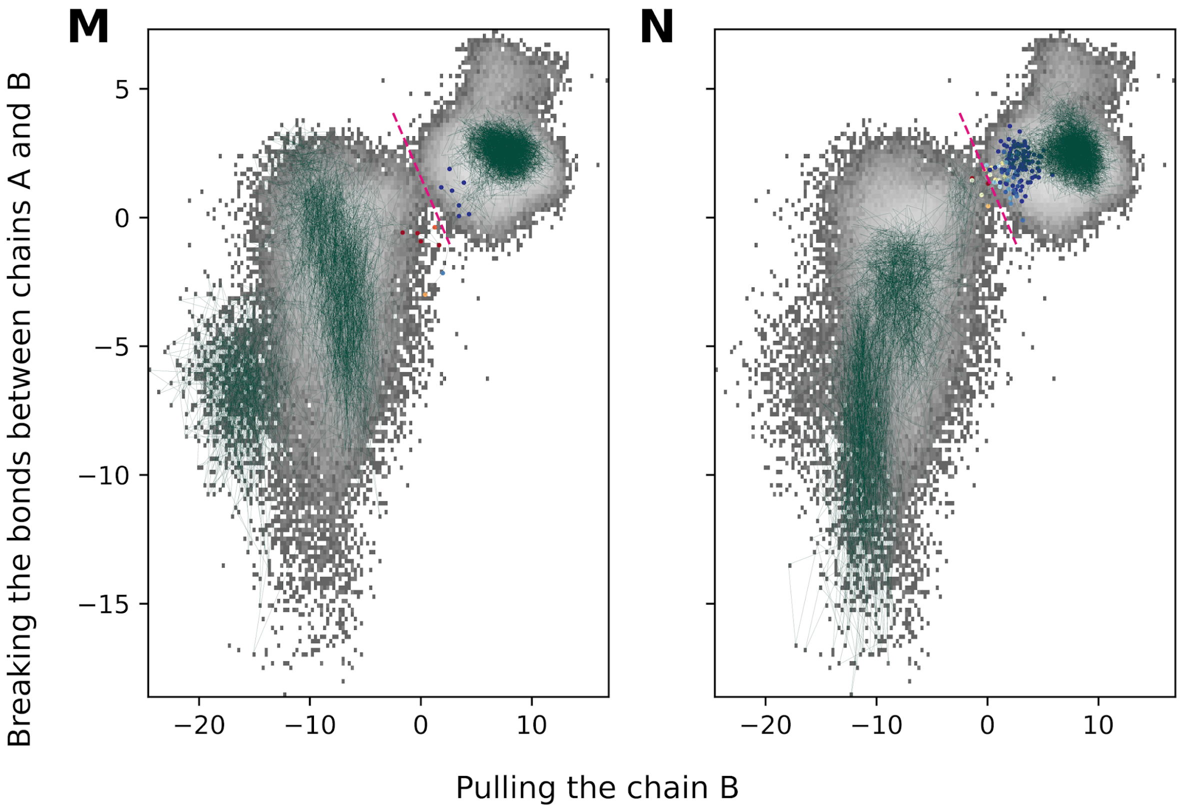

Figure S5. Continued. **M.** Replica 12a, **N.** Replica 12b.

Table S1. Changes in the contact sites during the transition from the closed to the S1 state

Sites of contacts	Location	The average frequency of the contacts in the site (Mean±SEM)			
		Closed state at zero tension	Closed state at tension	Transition state	S1 state
Site 1 ('periplasmic')	between the TM1 and TM2 helices of chain B	0.84±0.02	0.12±0.06	0.03±0.02	0.00±0.00
Site 2 ('cytoplasmic')	between the TM1 helices of chains A and B	0.85±0.04	0.60±0.09	0.26±0.09	0.07±0.05
Site 2 ('cytoplasmic')	between the TM1 helices of chains B and C	0.85±0.04	0.70±0.06	0.65±0.07	0.53±0.09
Site 3	between the N-terminal helix of chain B and the TM2 helix of chain D	0.88±0.04	0.82±0.02	0.79±0.01	0.77±0.03 (additionally new contacts are formed between N-terminal helix of chain B and F80, A83, and F84 residues of chain D)
Lipids in binding pocket	Acyl chains interacting with V22 residue of chain A and	0.43±0.07	0.17±0.10	0.03±0.01	0.00±0.00

	I82 and V86 of chain B				

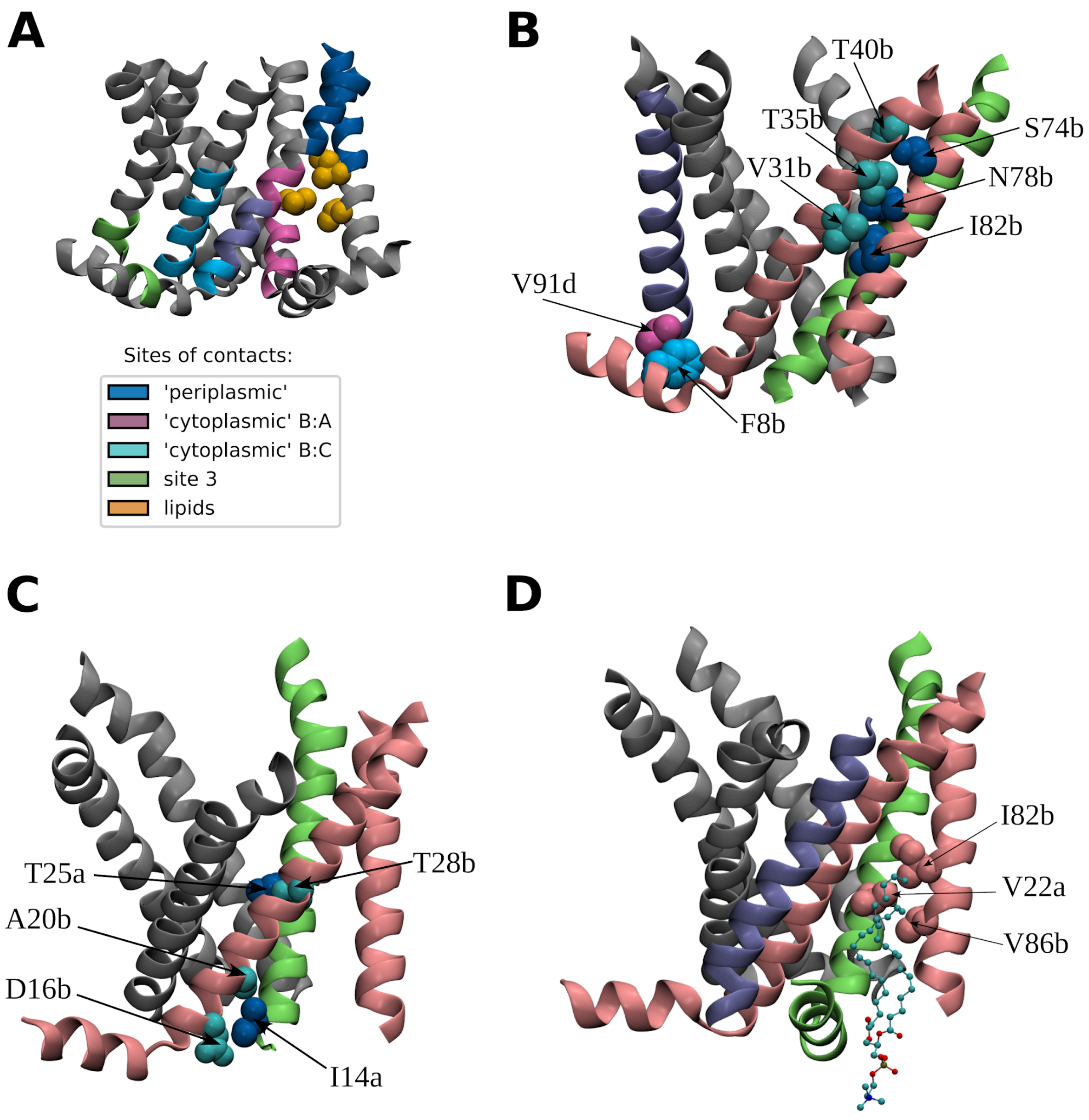

Figure S6. The closed state of MscL at zero tension. **A.** Relative position of all sites of contacts **B.** The 'periplasmic' contact site, represented by T40b:S74b, T35b:N78b, and V31b:I82b contacts, and the third site exemplified by the F8b:V91d contact. Chain B is highlighted in pink, the TM1 helix of chain A is shown in lime, the TM2 helix of chain D is highlighted in ice blue, and the remaining chains are shown in gray. **C.** The 'cytoplasmic' contact site between chains A

and B, represented by T25a:T28b, I14a:A20b and I14a:D16b contacts. The chains are colored in the same pattern as in panel b, but the TM2 helix of chain D is omitted. **D.** Lipid binding pocket formed by the I82 and V86 residues from chain B and the V22 residue from chain A in the closed state at zero tension. The lipid molecule occupying the pocket is shown with a ball-and-stick model.

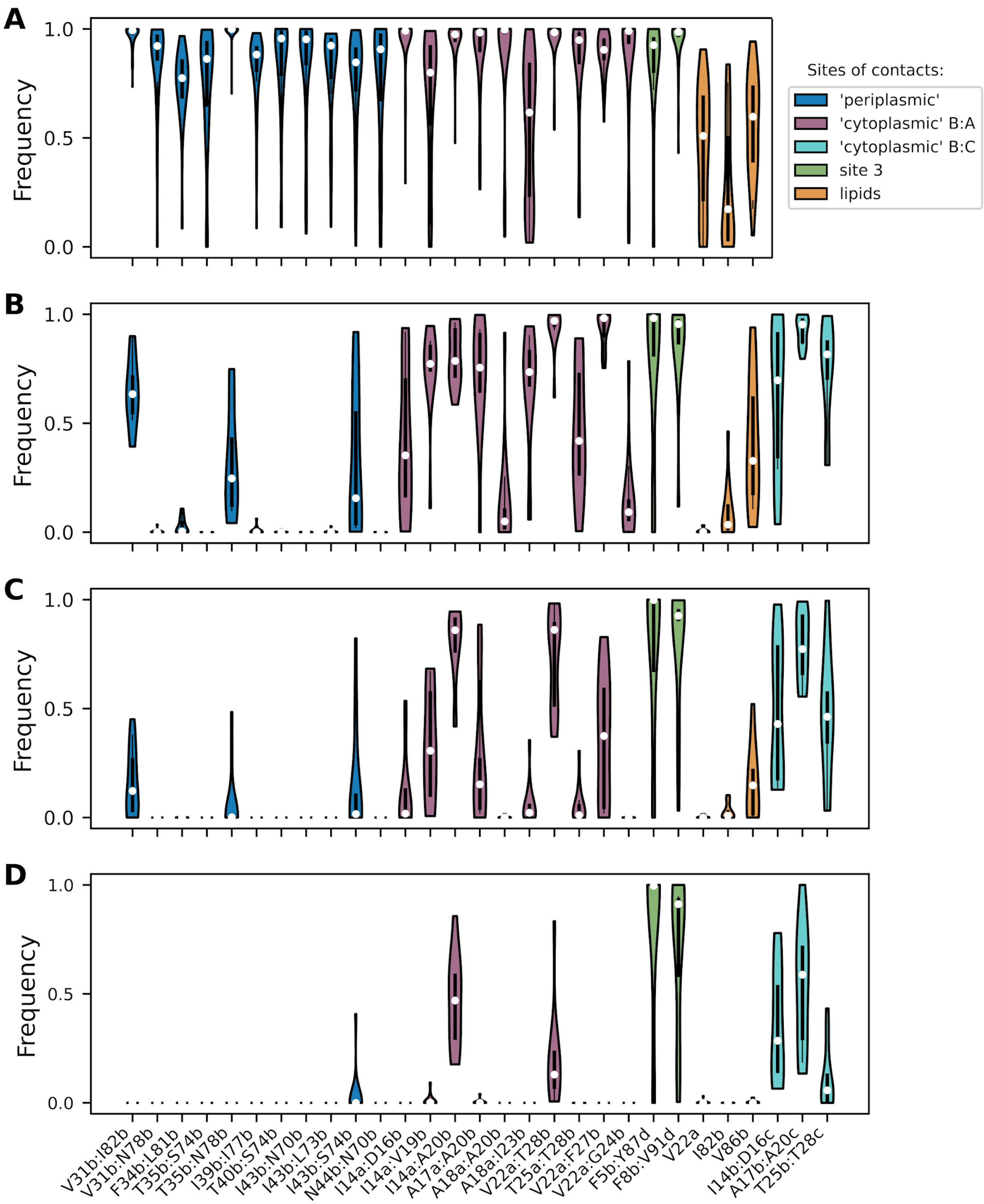

Figure S7. Frequencies of contacts stable in the closed state at zero tension during the transition of the chain B to the S1 state: **A.** closed state at zero tension, **B.** closed state under tension, **C.** transition state, defined as a range of the optimized collective variable from -5.4 to 2.4, **D.** S1

state. To plot the contact frequencies, two residues were considered to be in contact if the minimum distance between any two heavy atoms of the respective residues was less than 4.5 Å. The white circles represent the medians, the black thick lines extend from the first to the third quartile of the distributions.

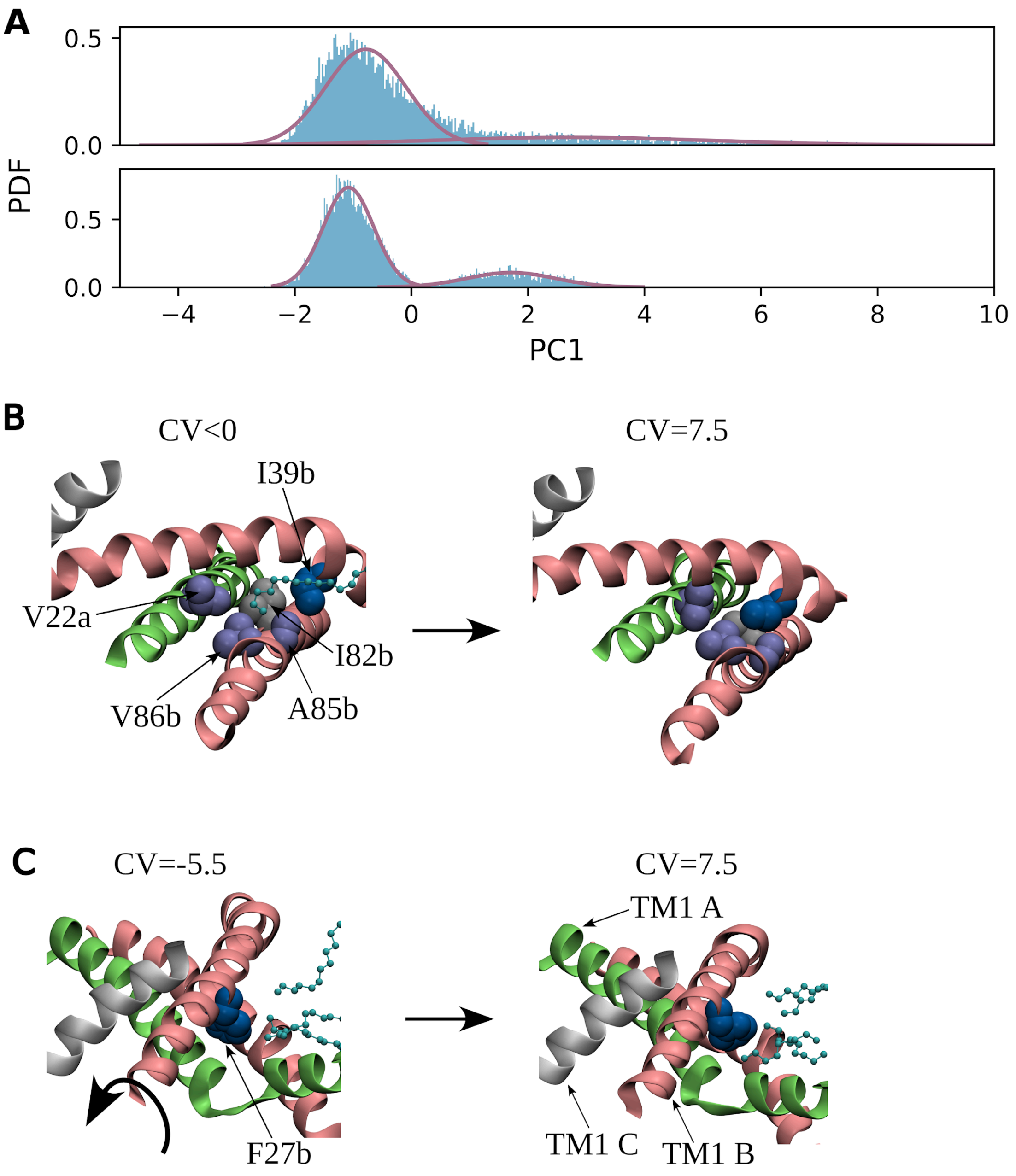

Figure S8. Impact of lipids on the transition of MscL to the S1 state. **A.** The probability density functions of two collective variables for Replica 7b. Both variables represent a first principle component calculated on a set of contacts. However, the top set comprises solely protein residues, whereas the bottom set incorporates additionally the contacts between lipids and the V22a:I82b:V86b binding pocket. The peak on the right corresponds to the closed state at tension, while the peak on the left corresponds to the S1 state. **B.** The left-hand representation illustrates the positioning of the lipid acyl chain within the V22a:I82b:V86b binding pocket in the closed state, with the applied tension. The right-hand representation depicts the I39 residue of the TM1 helix of chain B, which occupies the same pocket and forms contacts with the A85 and V86 residues in the S1 state. **C.** Upon reaching the S1 state, the F27 residue of chain B increases contacts with lipid acyl moieties, which is accompanied by a slight clockwise rotation of the TM1 helix.

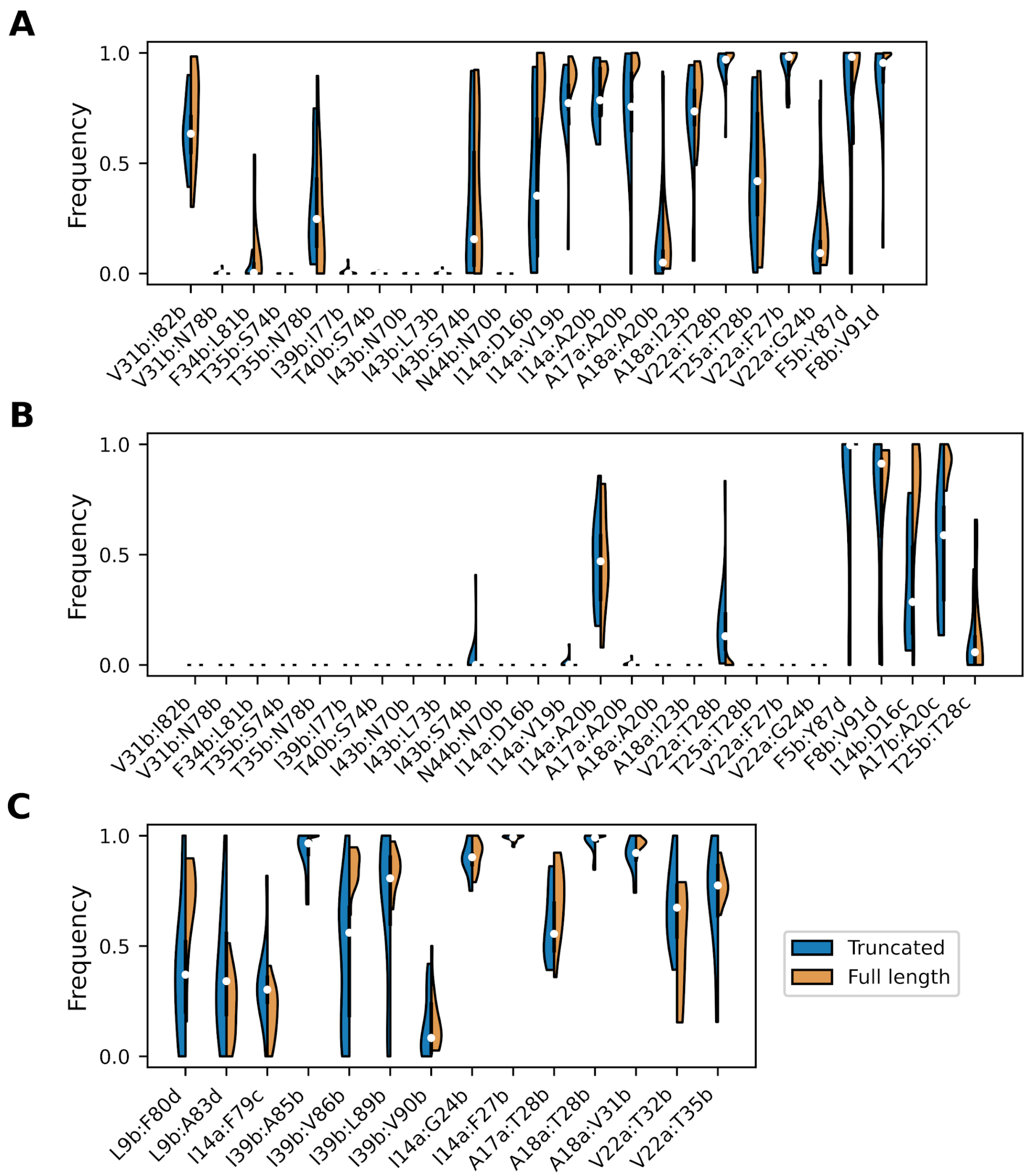

Figure S9. Frequencies of contacts in MscL with and without C-terminal domain **A.** closed state under tension, **B.** S1 state, **C.** S1 state. To plot the contact frequencies, two residues were considered to be in contact if the minimum distance between any two heavy atoms of the respective residues was less than 4.5 Å. The white circles represent the medians, the black thick lines extend from the first to the third quartile of the distributions.
